# Supplementary material for: Cryo-EM structures of MERS-CoV and SARS-CoV spike glycoproteins reveal the dynamic receptor binding domains
Source: Nat Commun. 2017 Apr 10;8:15092. doi: 10.1038/ncomms15092 (PMC5394239; doi:10.1038/ncomms15092)
Supplement: Supplementary Information — Supplementary Figures and Supplementary Tables [file ncomms15092-s1.pdf]

Supplementary Figures and Tables:

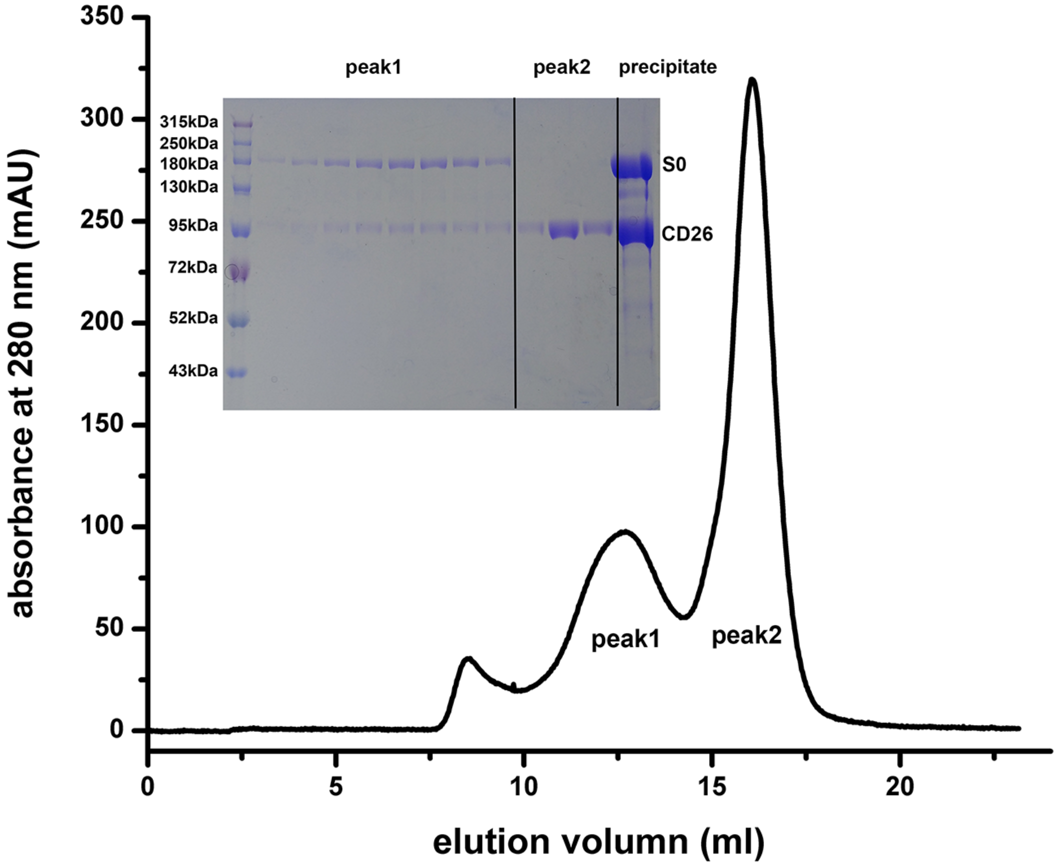

Supplementary Fig. 1 Gel filtration profile of uncleaved MERS-CoV S ectodomain trimer in complex with excessive CD26 protein. Most of the complex protein forms precipitant. MERS-CoV S trimer and CD26 can survive in the gel filtration, which is confirmed by the SDS-PAGE.

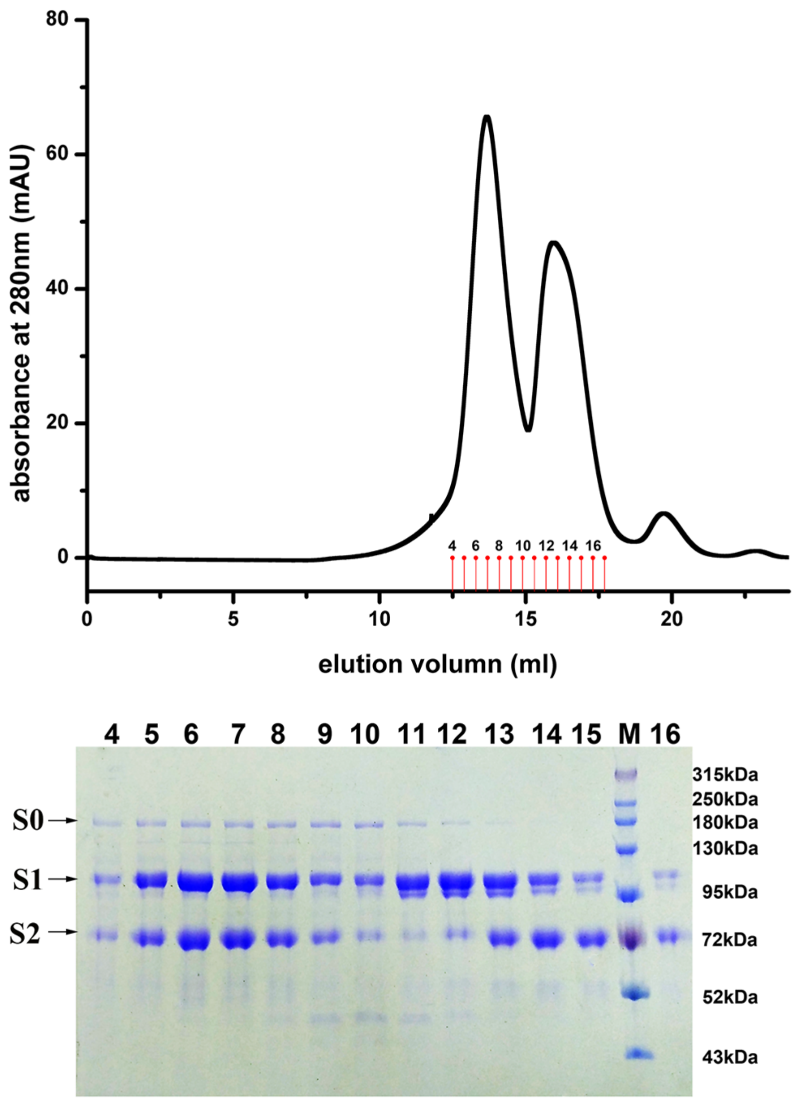

Supplementary Fig. 2 Gel filtration profile of cleaved MERS-CoV S ectodomain protein. There are two peaks in the gel filtration curve. One peak is the cleaved MERS-CoV S ectodomain trimer, and the other peak is a mixture of disassociated S1 and S2 subunits, confirmed by SDS-PAGE.

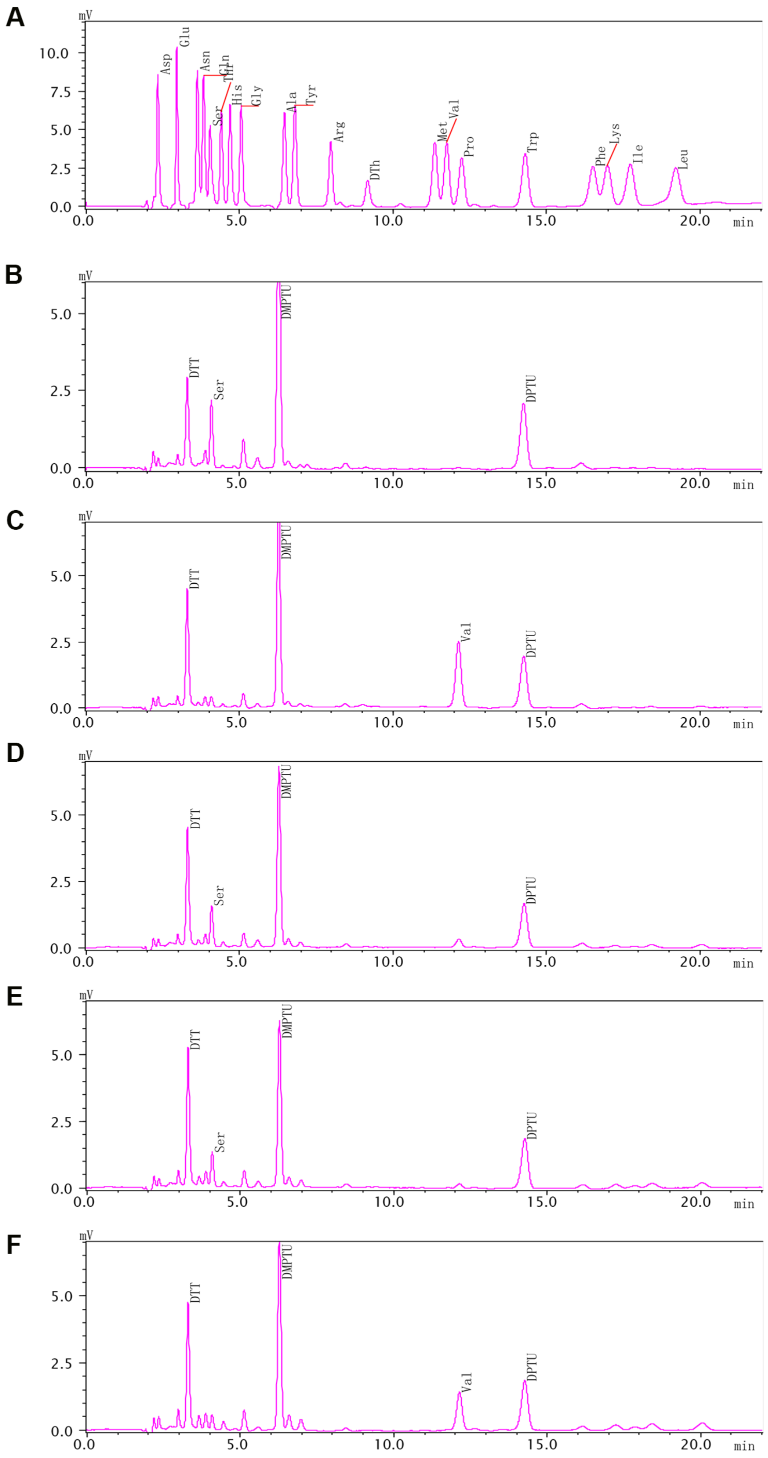

Supplementary Fig. 3 N-terminal amino acid sequencing of the cleaved S2 subunit. (A) Standard mass spectrometry map for different amino acids. (B-F) Mass spectrometry maps of the first five N-terminal amino acids of the S2 subunit band in Extended Data Figure 2. The maps show the first five amino acids are SVSSV.

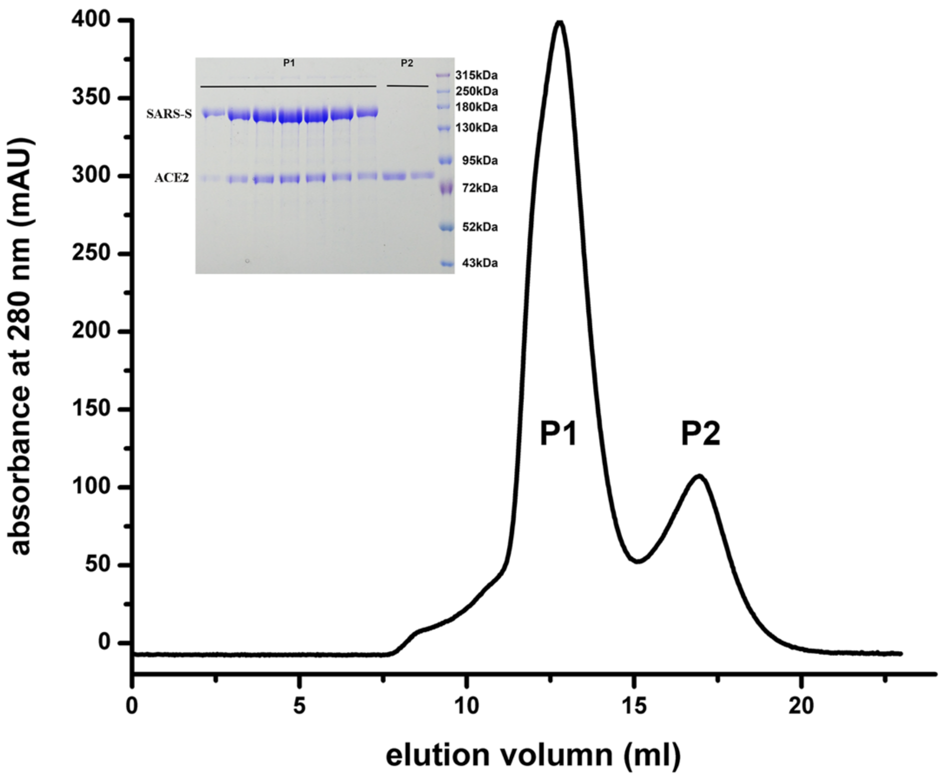

Supplementary Fig. 4 Gel filtration profile of uncleaved SARS-CoV S ectodomain trimer in complex with excessive ACE2 protein. SARS-CoV S trimer and ACE2 can survive in the gel filtration, which is confirmed by the SDS-PAGE.

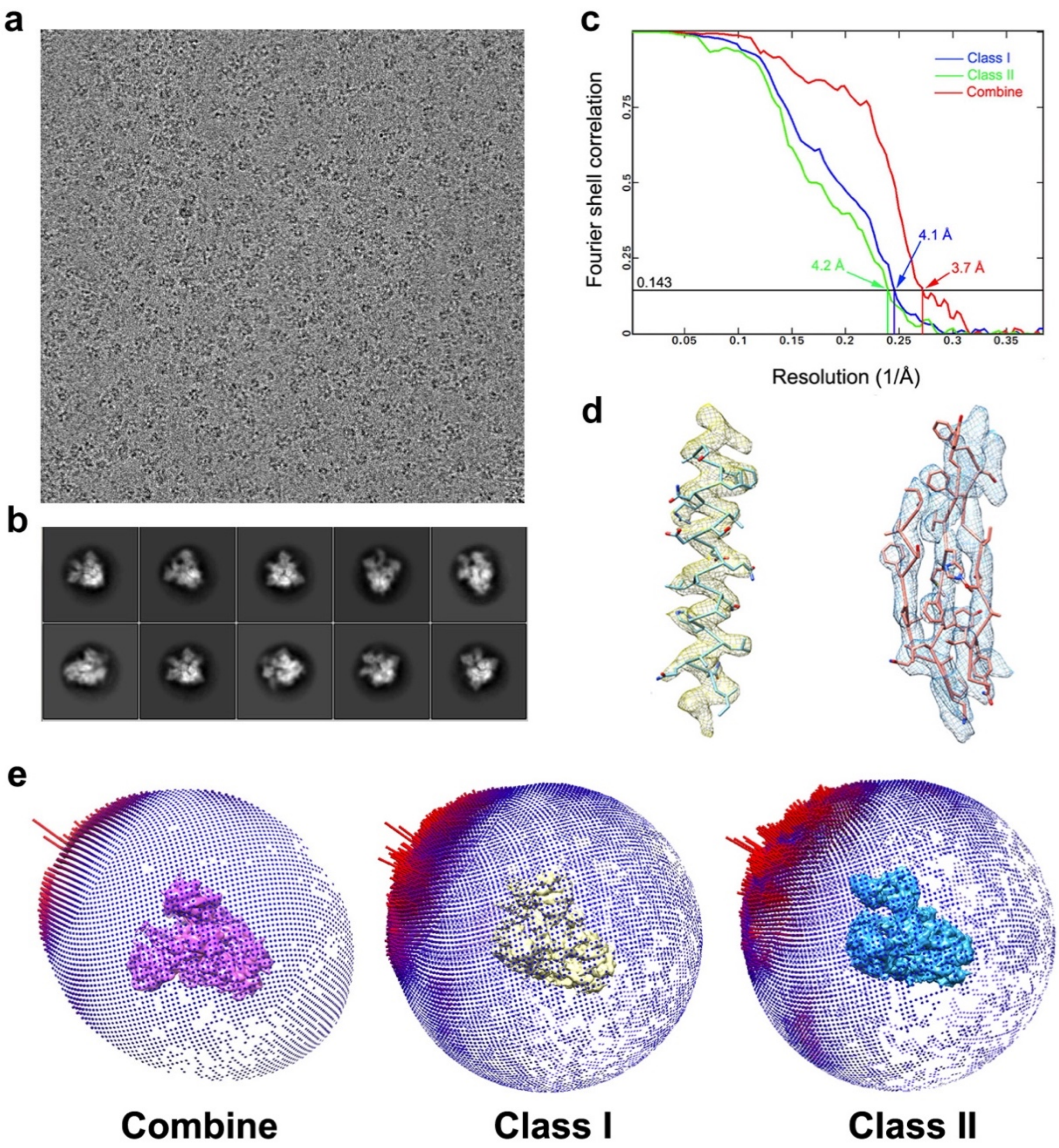

Supplementary Fig. 5 CryoEM analysis of the MERS-CoV S trimer. (**a, b**) Representative electron micrograph (defocus: 2.3μm) (**a**) and 2D class averages (**b**) of the MERS-CoV S trimer embedded in vitreous ice. (**c**) Angular distribution of the particlesused in the three-fold symmetric map and class one, class two asymmetric maps. (**d**) Gold-standard Fourier shell correlation (FSC) curves (three-fold symmetry: red, asymmetry: blue and green). The resolution of three-fold symmetric map and asymmetric maps (Class I and Class II) were determined to 3.7 Å, 4.1 Å and 4.2 Å, respectively. The 0.143 cut-off value is indicated by a horizontal blue line. (**e**) Cryo-EM density for selected regions (the central helix and β-hairpin) of MERS-CoV S reconstruction.

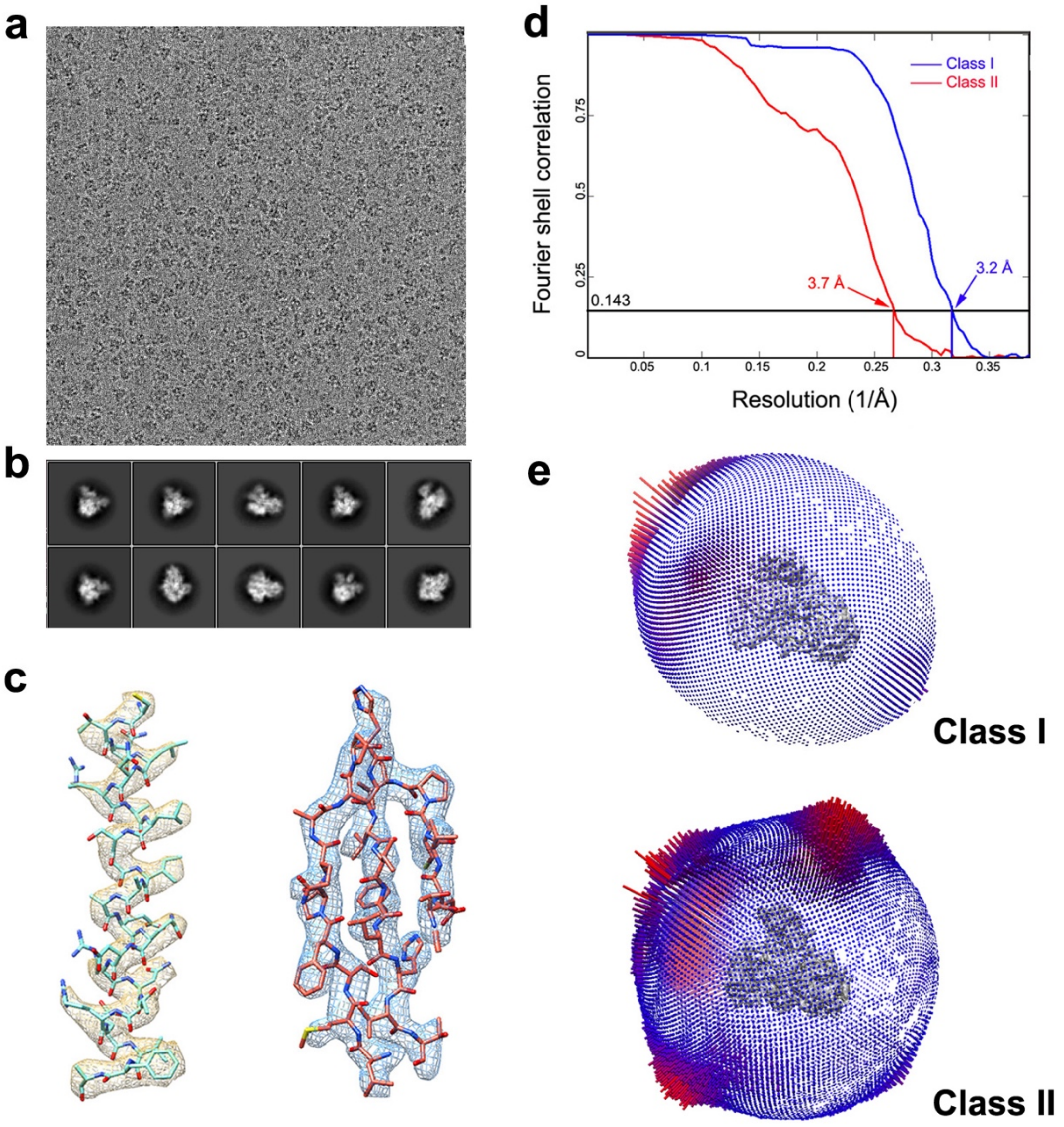

Supplementary Fig. 6 CryoEM analysis of the SARS-CoV S trimer.(**a, b**) Representative electron micrograph (defocus: 2.5μm) (**a**) and 2D class averages (**b**) of the MERS-CoV S trimer embedded in vitreous ice. (**c**) Angular distribution of the particles used in the three-fold symmetric map and asymmetric map. (**d**) Gold-standard Fourier shell correlation (FSC) curves (three-fold symmetry: blue, asymmetry: red). The resolution of three-fold symmetric map and asymmetric map (Class I and Class II) were determined to 3.2 Å and3.7 Å, respectively. The 0.143 cut-off value is indicated by horizontal blue line. (**e**) Cryo-EM density for selected regions (the central helix and β-hairpin) of SARS-CoV S reconstruction.

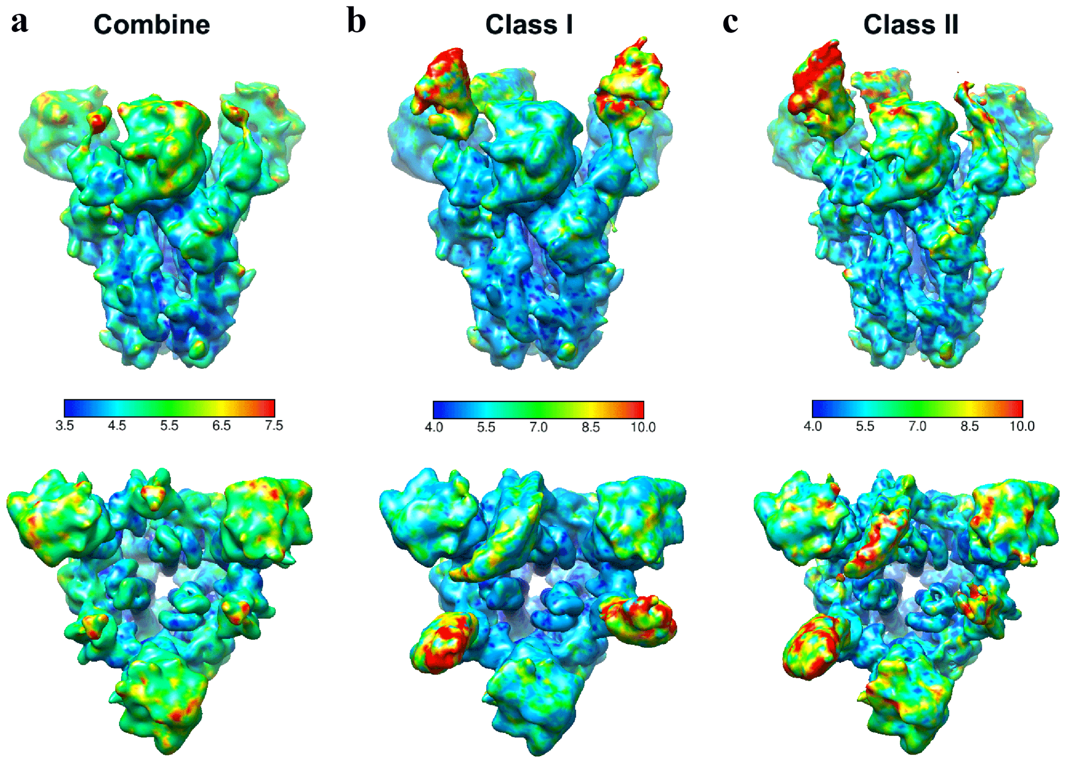

Supplementary Fig. 7 Local resolution maps of MERS-CoV S trimer. The three-fold symmetric map (**a**) and the asymmetric maps (**b and c)** of MERS-CoV S trimer from the side view and the top view, colored according to the resolution. The S2 part of the trimer has the resolution of ~ 3.5 Å and 4.0 Å for symmetric map and asymmetric maps, respectively. The "standing" RBD region has a resolution of ~ 7Å. The NTD and "lying" RBD regions have the resolution of ~ 4.5 Å.

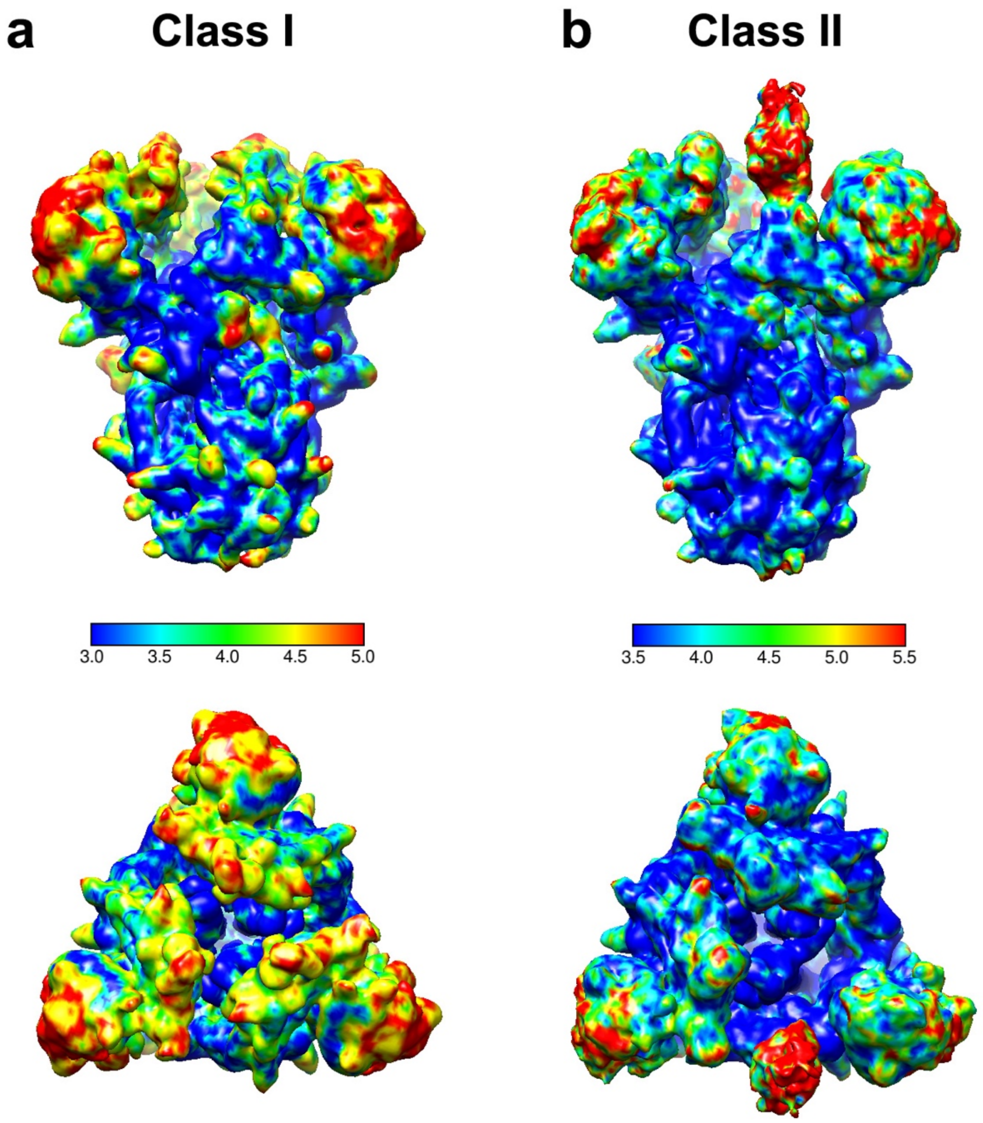

Supplementary Fig. 8 Local resolution maps of SARS-CoV S trimer. The three-fold symmetric map (**a**) and the asymmetric maps (**b and c)** of MERS-CoV S trimer from the side view and the top view, colored according to the resolution. The S2 part of the trimer has the resolution of ~ 3.0 Å and 3.5 Å for symmetric map and asymmetric map, respectively. The "standing" RBD region has a resolution of ~ 5Å. The NTD and "lying" RBD regions have the resolution of ~ 4Å.

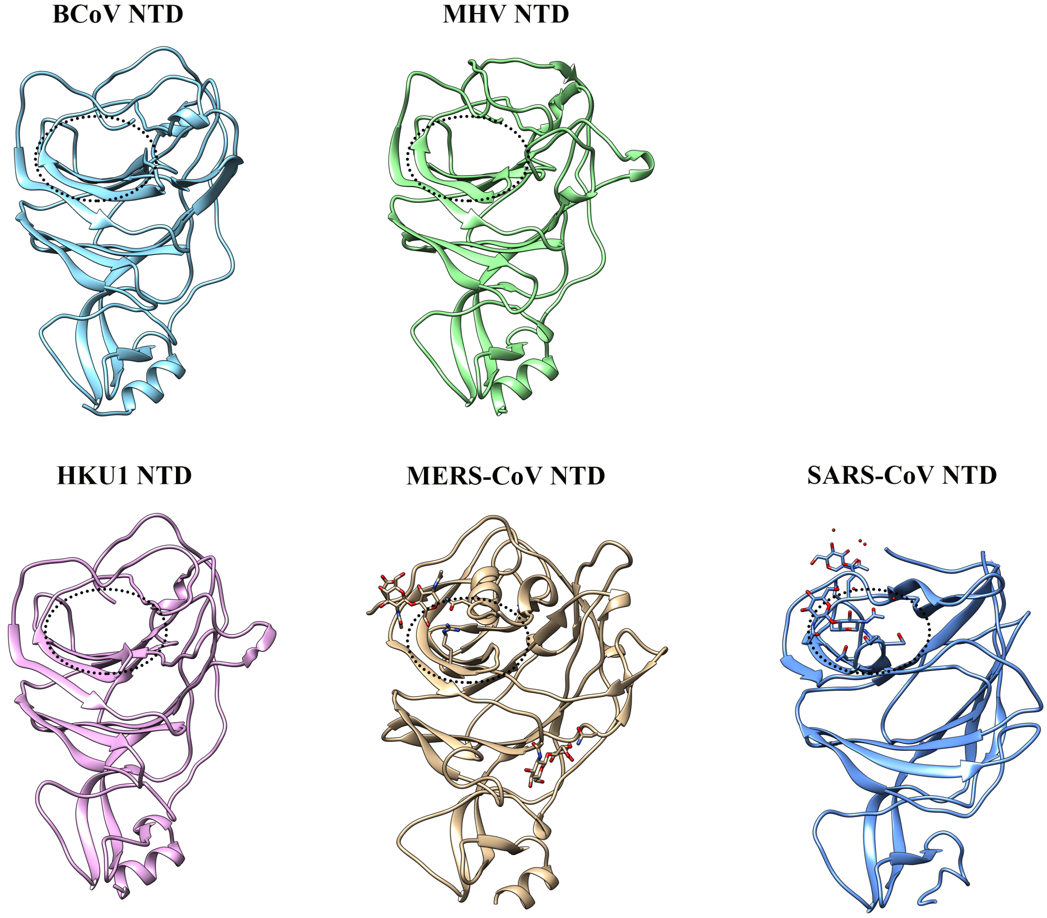

Supplementary Fig. 9 Comparison of NTD structures from BCoV, MHV, HKU1 SARS-CoV and MERS-CoV. MERS-CoV and SARS-CoV NTD structures fold into a galectin-like structure as in bovine coronavirus (BCoV), MHV and HKU1. However, the glycan binding site in MERS-CoV and SARS-CoV NTDs are occupied by a short helix or the glycans, preventing it to bind to host glycans. The glycan binding site are marked by dashed circles.

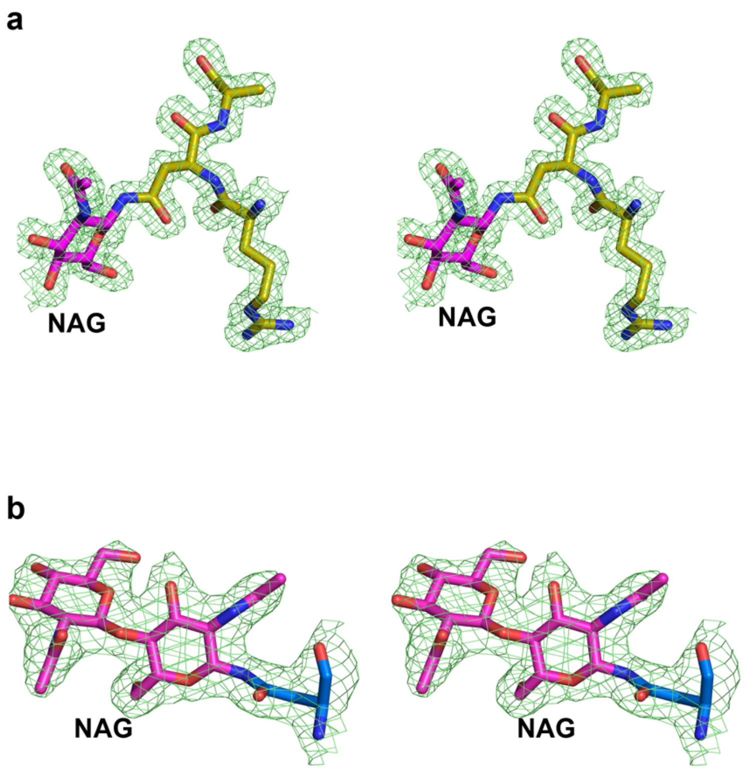

Supplementary Fig. 10 Stereo views of crystal structures from MERS-CoV and SARS-CoV NTDs. The 2Fo-Fc omit maps contoured at 1 σ of the glycosylation sites in MERS-CoV (a) and SARS-CoV (b) NTDs are shown as green mesh. The glycans are shown in magenta sticks.

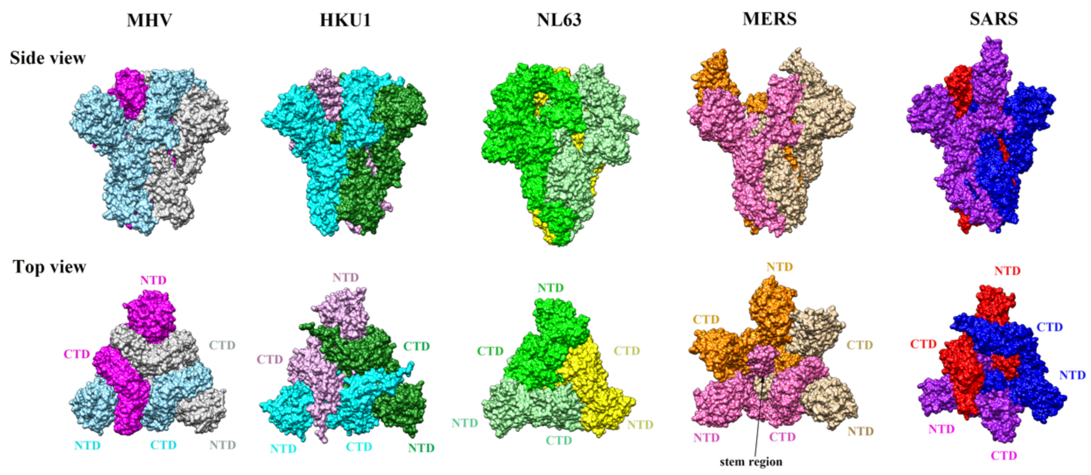

Supplementary Fig. 11 Comparison of MHV, HKU1, NL63, SARS-CoV and MERS-CoV S trimer structures. Only MERS-CoV S trimer shows an open conformation of S1 subunit, whereas the MHV, HKU1 and NL63 S trimers show a closed conformation of S1 subunit.

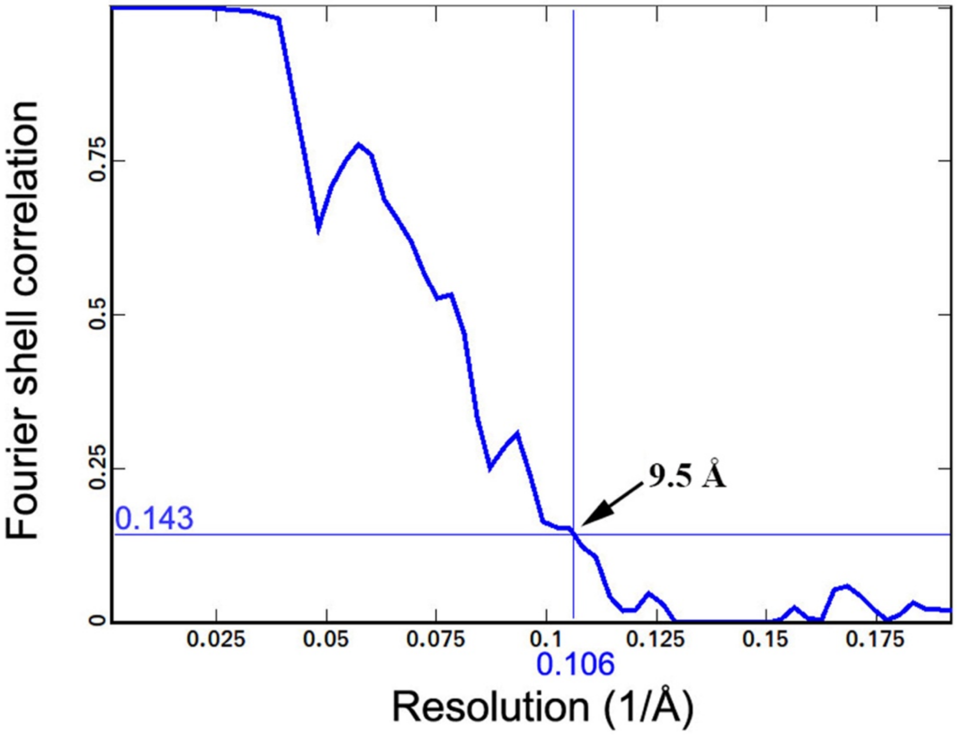

Supplementary Fig. 12 Gold-standard (blue) Fourier shell correlation (FSC) curve of disassociated S1 trimer. The resolution was determined to 9.5 Å. The 0.143 cut-off value is indicated by a horizontal blue line.

Supplementary Table 1. Crystallographic data collection and refinement statistics of the MERS-CoV NTD and SARS-CoV NTD.

	MERS-NTD (KAuCl4)	MERS-NTD	SARS-NTD
**Data collection**			
Space group	P212121	P212121	P6122
Cell dimensions			
a, *b*, *c* (Å)	52.81, 85.50, 88.24	51.65, 86.61, 88.10	73.29, 73.29, 240.26
α, β, γ (°)	90.00, 90.00, 90.00	90.00, 90.00, 90.00	90.00, 90.00, 120.00
Resolution (Å)	50.00-2.00 (2.07-2.00)*	50-1.50 (1.55-1.50)	50-2.20 (2.28-2.20)
R_{merge}	0.067 (0.625)	0.073 (1.163)	0.135 (0.548)
$I / \sigma I$	37.5 (5.3)	27.6 (1.6)	14.6 (5.9)
Completeness (%)	99.5 (97.5)	99.7 (99.7)	99.4 (100.0)
Redundancy	13.4 (11.7)	8.4 (8.4)	11.1 (14.2)
**Refinement**			
Resolution (Å)		44.34-1.50	38.31-2.20
No. reflections		60850	20247
R_{work} / R_{free}		0.1625/0.1946	0.2520/0.2826
No. atoms			
Protein		2718	2194 -
Ligand/ion		-	84
Water		356	
B-factors ($Å^2$)			
Protein		16.2	54.57
Ligand/ion		-	
Water		31.7	56.37
R.m.s. deviations			
Bond lengths (Å)		0.004	0.003
Bond angles (°)		0.958	0.74
Favored		96.44	93
Allowed		3.56	7.2
Outliers		0	0

*Highest resolution shell is shown in parenthesis.

Supplementary Table 2. Statistics of the structural models of the MERS-CoV and SARS-CoV S trimers refined against the 3.7 Å and 3.2 Å resolution cryo-EM maps.

Model statistics	MERS-CoV S	SARS-CoV S
FSCaverage	0.7513	0.8864
Rfactor overall	0.3641	0.3254
R.m.s. deviations		
Bond length (Å)	0.0130	0.01
Bond angles (°)	2.0652	1.2
Ramachandran statistics (%)		
Favored regions	78.97	91.92
Allowed regions	19.72	8.08
Outlier regions	1.31	0.00
Rotamer outlier (%)	2.34	0.29
Clash score	12.40	4.77
